# Supplementary material for: Increased Inflammation and Cardiometabolic Risk in Individuals with Low AMY1 Copy Numbers
Source: J Clin Med. 2019 Mar 19;8(3):382. doi: 10.3390/jcm8030382 (PMC6463254; doi:10.3390/jcm8030382)
Supplement: Supplementary file 1 [file jcm-08-00382-s001.pdf]

MS: JCM-454190: Increased inflammation and cardiometabolic risk in individuals with low AMY1 copy numbers

Supplementary Table 1. Daily macronutrient intake of low and high *AMY1* CNV groups.

**Macronutrient**	**Low *AMY1* (n=29)**	**High *AMY1* (n=28)**	***P***
Starch (g/day)	145.5 ± 43.6	150.3 ± 59.6	0.8
Protein (g/day)	92.6 ± 22.8	89.9 ± 46.5	0.8
Total Fat (g/day)	83.5 ± 24.2	68.9 ± 32.7	0.1
Saturated Fat (g/day)	30.6 ± 10.7	24.8 ± 12.8	0.1
Total Energy (kJ/day)	8792.2 ± 2224.3	8016.89 ± 2897.0	0.3

Data are expressed as mean ± SD. Low *AMY1* group is defined as ≤4 copies and high AMY1 group as >4 copies. Differences between groups were analysed using independent Student's *t*-tests. Variables which did not fit a normal distribution were log-transformed to the base 10 to approximate normality prior to analysis.
